# Application of rubber band and clip traction for removal of a submucosal fecalith mimicking a submucosal tumor of the appendix under colonoscopy

**DOI:** 10.1055/a-2106-0768

**Published:** 2023-06-22

**Authors:** Longping Chen, Linfu Zheng, Zhiping Chen, Dazhou Li, Wen Wang

**Affiliations:** 1 Department of Gastroenterology, Fuzhou General Clinical Medical College, Fujian Medical University, Fuzhou, China; 2 Department of Gastroenterology, 900th Hospital of People’s Liberation Army, Fuzhou, China; 3 Department of Gastroenterology, Oriental Hospital Affiliated to Xiamen University, Fuzhou, China

The term “submucosal tumors” generally refers to colorectal uplifted lesions with a complete upper mucosal cortex, including lipomas, stromal tumors, lymphomas, and neuroendocrine tumors 1 . Approximately 13 % of gastrointestinal submucosal tumors are malignant. Endoscopic ultrasonography (EUS) is the first choice of modality for the diagnosis of submucosal tumors 2 . Here we report an unusual case of a submucosal fecalith mimicking a submucosal tumor of the appendix.

A 74-year-old woman who had been experiencing right lower abdominal pain for more than 3 months was admitted to our hospital for evaluation of a submucosal “tumor” of the appendix that had been identified 1 month previously. It has been difficult to histologically diagnose subepithelial abnormalities of the appendix by means of endoscopic biopsy. However endoscopic ultrasound (EUS) can be used to complete the scanning of appendiceal lesions, and this has been of great significance in the observation of subepithelial appendiceal lesions. A linear-array echoendoscope was placed at the opening of the appendix. By means of the ultrasound-guided mirror insertion method, a raised lesion was observed in the appendix, with a section size of 14.3 × 8.9 mm, raising the possibility that the appendiceal lesion might be a submucosal fecalith ( Fig. 1 ).

**Fig. 1 a:**
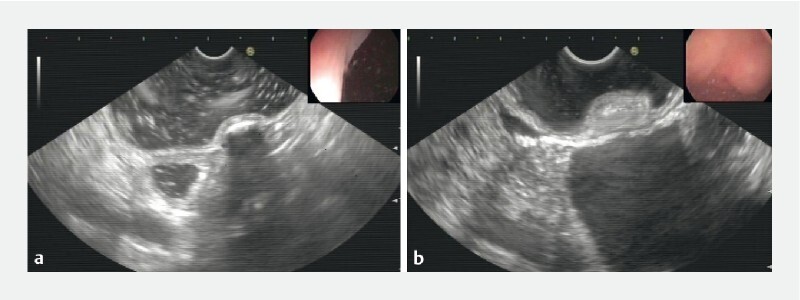
Endoscopic ultrasonography showed that the submucosal appendiceal lesion had clear boundaries with a section size of 14.3 × 8.9 mm, and the acoustic shadow was visible behind. **b** Endoscopic ultrasonography showed that the lesion originated from the third layer and mainly grew into the cavity, with low and uneven echoes and a high internal echo.

Although laparoscopic appendectomy has always been the main treatment option for acute appendicitis caused by appendiceal fecaliths, Salminen et al. showed that the complication rate associated with this surgery could be as high as 20 % 3 . With the gradual development of endoscopic technology, endoscopic appendectomy lithotomy has also been occasionally reported 4 . We therefore employed endoscopic submucosal dissection, aided by traction applied using a clip with rubber band, to remove the appendiceal submucosal fecalith. This improved the accuracy of the endoscopic appendectomy in removing the fecalith and avoided damage to the intestinal wall ( Fig. 2 , Video 1 ). Finally the patient recovered well and was discharged, and showed no abdominal pain during follow-up.

**Fig. 2 a:**
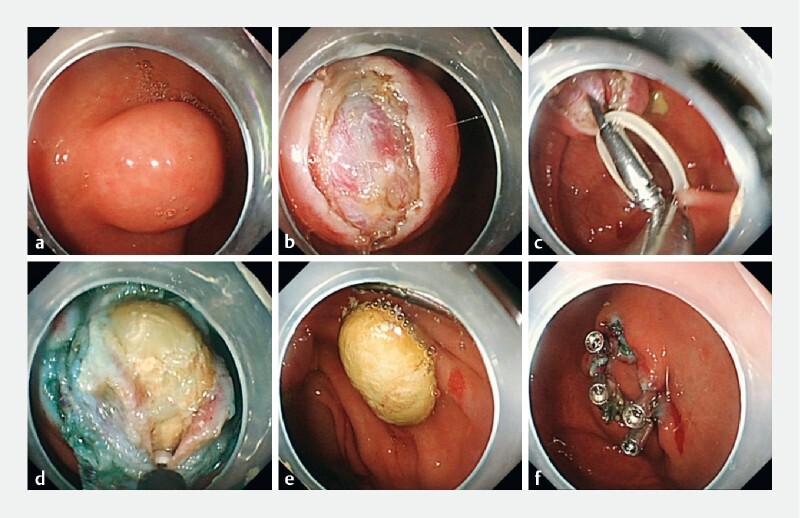
Colonoscopy revealed a submucosal lesion approximately 1.0 × 1.2 cm in size at the appendix. **b** The surface mucosa was dissected endoscopically. **c** Clip and rubber band and traction. **d** Incision of the submucosa and muscular layer exposed a gold-colored fecal stone in the appendix. **e** The oval-shaped appendiceal fecal stone. **f** The wound was closed with clips to reconstruct the appendiceal orifice.

**Video 1** A linear-array endoscopic ultrasound (EUS) device is inserted into the appendix and endoscopic submucosal dissection is performed, aided by the clip and rubber band traction technique, to remove an appendiceal fecalith.


Submucosal fecaliths mimicking submucosal tumors of the appendix have been rarely reported in clinical practice. Preoperative EUS is extremely important for auxiliary evaluation of the nature of submucosal tumors. Endoscopic appendectomy for stone removal is a safe, effective, and minimally invasive method for diagnosis and treatment in such cases.

Endoscopy_UCTN_Code_TTT_1AQ_2AD

## Notes

Endoscopy E-Videos

https://eref.thieme.de/e-videos
*E-Videos* is an open access online section of the journal *Endoscopy* , reporting on interesting cases and new techniques in gastroenterological endoscopy. All papers include a high-quality video and are published with a Creativse Commons CC-BY license. Endoscopy E-Videos qualify for HINARI discounts and waivers and eligibility is automatically checked during the submission process. We grant 100% waivers to articles whose corresponding authors are based in Group A countries and 50% waivers to those who are based in Group B countries as classified by Research4Life (see: https://www.research4life.org/access/eligibility/ ). This section has its own submission website at https://mc.manuscriptcentral.com/e-videos
